# Eco-Friendly Synthesis and Characterization of *Senna italica*–Derived Silver Nanoparticles With Broad-Spectrum Antimicrobial Activity

**DOI:** 10.1155/ijm/2072594

**Published:** 2025-05-08

**Authors:** Emad Abada, Fatimah Habib, Abdullah Mashraqi, Yosra Modafer, Wail Alsolami, Khatib Ismail, Abdullah Ali Alamri, Abadi M. Mashlawi, Abdel-Rahman M. Shater

**Affiliations:** ^1^Department of Biology, College of Science, Jazan University, P.O. Box 114, Jazan 45142, Saudi Arabia; ^2^Environment and Nature Research Center, Jazan University, P.O. Box 114, Jazan 45142, Saudi Arabia; ^3^Department of Physical Sciences, Chemistry Division, College of Science, Jazan University, P.O. Box 114, Jazan 45142, Saudi Arabia; ^4^Nanotechnology Research Unit, College of Science, Jazan University, P.O. Box 114, Jazan 45142, Saudi Arabia

**Keywords:** antimicrobial, leaf extract, MIC, *Senna italica*, silver nanoparticles

## Abstract

The eco-friendly and cost-effective biological synthesis of nanomaterials is rapidly gaining attention. This study synthesized silver nanoparticles (AgNPs) using an aqueous extract of *Senna italica* leaves and silver nitrate (AgNO_3_). The synthesized AgNPs were characterized using UV-Vis spectroscopy, Fourier-transform infrared (FTIR) spectroscopy, transmission electron microscopy (TEM), scanning electron microscopy (SEM), and X-ray diffraction (XRD). UV-Vis spectroscopy confirmed the formation of AgNPs, displaying a characteristic surface plasmon resonance peak at 445 nm. TEM and SEM analyses revealed spherical nanoparticles with sizes ranging from 12.7 to 24 nm. FTIR spectra identified bands at 1636 and 3496 cm^−1^, corresponding to C=O and O-H groups, indicating their role in stabilizing the nanoparticles. XRD analysis revealed diffraction planes at 111, 200, 220, and 311, consistent with the face-centered cubic structure of silver. The AgNPs demonstrated significant antimicrobial activity against fungi and Gram-negative and Gram-positive bacteria, with *Escherichia coli* showing the highest sensitivity (MIC = 0.014*  μ*g/mL). SEM analysis of *E. coli* showed that untreated cells retained their normal morphology, whereas AgNP-treated cells appeared shriveled and deformed. These results underscore the potential of *Senna italica*–derived AgNPs as effective antimicrobial agents. Future studies will be aimed at investigating the detailed mechanisms underlying the effects of AgNPs on bacterial cell structure and growth.

## 1. Introduction

The synthesis of silver nanoparticles (AgNPs) from plants represents a fascinating and rapidly growing area of research, mainly due to these nanoparticles' highly effective antimicrobial properties [1]. The fundamental process of synthesizing AgNPs using plants typically involves the extraction of active substances from various plant parts, such as leaves, roots, or flowers, and subsequently converting these substances into AgNPs. This method has emerged as a promising alternative to conventional chemical synthesis due to its simplicity, cost-effectiveness, and environmental compatibility [2]. In recent years, there has been a significant surge in interest in developing and discovering new antibacterial agents to combat the growing global threat of antimicrobial resistance. Antimicrobial resistance has become a pressing concern, primarily driven by the widespread misuse and overuse of antibiotics in human medicine, veterinary medicine, and agriculture [3]. This has resulted in the evolution of bacterial strains resistant to many currently available drugs. The need for innovative and effective antimicrobial agents has never been more urgent, and AgNPs offer a promising solution [4].

AgNPs, ranging in size from 1 to 100 nm, are among the most extensively studied due to their unique and distinctive properties. Their small size and high surface-area-to-volume ratio confer exceptional biological and chemical activities, making them highly effective against various fungi and bacteria [5]. These nanoparticles are widely utilized in diverse fields, including agriculture, medicine, and consumer products. Their versatility stems from their antimicrobial, antiviral, and anti-inflammatory properties and their ability to incorporate into various applications when synthesized and utilized appropriately and safely [6].

However, the conventional chemical synthesis of AgNPs often has significant drawbacks, including high costs and environmental toxicity associated with using hazardous capping agents and reducing agents. To overcome these challenges, researchers have turned to green synthesis, an eco-friendly and cost-effective approach that leverages plant-based materials for the synthesis of nanoparticles. This method is nontoxic, sustainable, and capable of overcoming many of the limitations of earlier techniques. Furthermore, green synthesis is more straightforward and less expensive than microorganism-based synthesis methods [7].

The green synthesis process involves reducing silver nitrate (AgNO_3_) into AgNPs, facilitated by bioactive compounds such as flavonoids, phenols, and terpenoids in plant extracts. These compounds serve as reducing agents and stabilize the nanoparticles, ensuring their efficiency and safety. Several plant parts, including roots, flowers, leaves, and fruits, have been successfully employed for the rapid and efficient extracellular synthesis of AgNPs, demonstrating the versatility and potential of this approach [8].

Among the various plant species studied for nanoparticle synthesis, *Senna italica*, commonly known as Port Royal senna, *Italian senna*, or Senegal senna, stands out as a promising candidate. *Senna italica* is a legume tree belonging to the genus *Senna* [9]. It is a deciduous, perennial herb or shrub that grows up to 60 cm tall and is characterized by its woody structure. Despite the extensive research on AgNP synthesis from plant extracts, there have been very few reports on using *Senna* species for this purpose [10]. While studies have documented the synthesis of AgNPs from *Senna alata* and *Senna siamea*, using *Senna italica* for AgNP synthesis remains largely unexplored.

The primary aim of this study is to synthesize AgNPs using a green, environmentally friendly approach with aqueous leaf extracts of *Senna italica* and to evaluate their antimicrobial activity against a range of microorganisms. Advanced analytical techniques such as UV-Vis spectroscopy, Fourier-transform infrared (FTIR) spectroscopy, transmission electron microscopy (TEM), scanning electron microscopy (SEM), and X-ray diffraction (XRD) are employed to confirm the successful synthesis of AgNPs and to determine their structural and morphological properties. The study further assesses the antimicrobial efficacy of *Senna italica*–derived AgNPs against various pathogens, including fungi, Gram-negative bacteria, and Gram-positive bacteria, with a particular focus on understanding their specific mechanisms of action. Additionally, the role of bioactive compounds in *Senna italica* leaf extract, such as flavonoids, terpenoids, and phenols, is investigated to elucidate their role in reducing and stabilizing AgNPs. Moreover, the effects of synthesized AgNPs on bacterial morphology and growth are examined in detail to gain deeper insights into their interaction with bacterial cell structures. This study highlights the innovative use of *Senna italica* for the green synthesis of AgNPs and demonstrates their potential as effective antimicrobial agents, providing valuable insights into combating antimicrobial resistance.

## 2. Materials and Methods

### 2.1. Collection of Plant Samples and Preparation

The leaves of *Senna italica* were collected from the Jazan region, KSA (16°53⁣′21.69⁣^″^N). Dr. Ramesh, Head of the Plant Herbarium at the Department of Biology, College of Science, Jazan University, verified the plant's identification. To ensure cleanliness, the leaves were thoroughly washed with distilled water to remove dust or impurities. They were left to dry in a shaded, well-ventilated area at 27°C–29°C for 1 week. Once completely dried, the leaves were finely ground using an electric grinder to obtain a uniform powder, which was subsequently used for further analysis.

### 2.2. Preparation of Plant Extract

Ten grams of finely ground *Senna italica* leaf powder was added to 100 mL of distilled water and heated to 80°C for 20 min under continuous stirring to ensure proper extraction of bioactive compounds. The resulting mixture was then filtered using No. 6 filter paper to remove residual plant material. To further purify the extract, the filtrate was centrifuged at 8000 rpm for 10 min at 4°C. The supernatant, containing the active plant-derived compounds, was carefully transferred to a clean glass container and used as a natural reducing agent to synthesize AgNPs [11, 12].

### 2.3. Optimization of the Synthesis of Nanoparticles

The optimal conditions for synthesizing AgNPs from AgNO_3_ were systematically examined, including temperature, pH, the volume of plant extract, and the concentration of the AgNO_3_ solution. The mixture of *Senna italica* leaf extract and AgNO_3_ was continuously stirred using a magnetic stirrer for at least 2 h. During this process, a noticeable color change to brown was observed, indicating the successful formation of AgNPs [13].

#### 2.3.1. Effect of Different AgNO_3_ Concentrations

Various concentrations of AgNO_3_ solution were evaluated, specifically 3, 5, 7, 9, and 10 mM. Equal volumes of each concentration were mixed with an equal volume of *Senna italica* leaf extract and incubated at 60°C for 24 h. Following the incubation period, the formation of AgNPs was assessed using UV-Vis spectrophotometry to confirm their successful synthesis and analyze their optical properties.

#### 2.3.2. Effect of Different Dilutions of the Plant Extract

Several concentrations of *Senna italica* leaf extract solution were prepared in varying ratios (1:1, 1:2, 1:4, 1:10, 1:20, 1:40, 1:50, and 1:100). Equal volumes of each concentration were then mixed with an equal volume of AgNO_3_ solution under continuous stirring and incubated at 60°C for 24 h. The successful synthesis of AgNPs was subsequently confirmed through UV-Vis spectrophotometric analysis, which assessed the characteristic surface plasmon resonance of the nanoparticles.

#### 2.3.3. Effect of Temperatures

To investigate the effect of temperature on the formation of AgNPs, the reaction between *Senna italica* leaf extract and AgNO_3_ solution was conducted at various temperatures, including 4°C, 20°C, 37°C, 45°C, 60°C, and 70°C.

#### 2.3.4. Effect of Extract Volume

To examine the effect of leaf extract volume on the formation of AgNPs, varying volumes of *Senna italica* leaf extract (1, 2, 3, 4, 5, and 6 mL) were utilized in the reaction with AgNO_3_ solution.

#### 2.3.5. Effect of pH

To investigate the effect of pH on the synthesis of AgNPs, the reaction was conducted at varying pH levels, specifically 2, 4, 7, 10, 12, and 14.2.4.

### 2.4. Characterization of the AgNPs

#### 2.4.1. UV-Visible Spectroscopy Analysis

The synthesis of AgNPs was confirmed using a UV-visible spectrophotometer, which measured absorbance within the wavelength range of 200–1000 nm. The characteristic surface plasmon resonance peak of AgNPs was used to indicate successful nanoparticle formation [14].

#### 2.4.2. TEM Analysis

TEM was performed to analyze the size, shape, and morphology of the synthesized AgNPs. The analysis was conducted using a HITACHI H-800 (Japan) TEM at an accelerating voltage of 200 kV, following the methodology described in previous studies [15].

#### 2.4.3. FTIR Spectroscopy Analysis

FTIR spectroscopy was utilized to identify the functional groups present in the *Senna italica* leaf extract, which play a role in the reduction and stabilization of AgNPs. The analysis was performed using an FTIR spectrometer (Thermo Fisher Scientific, United States) over a 500–4000 cm^−1^ spectral range, employing a KBr pellet technique. The recorded spectra were plotted as transmittance (percentage) versus wavenumber (reciprocal centimeters) to determine the key functional groups involved in nanoparticle synthesis [16].

#### 2.4.4. XRD Analysis

XRD analysis was performed following the methodology outlined in [17] to determine the crystalline structure and phase composition of the synthesized AgNPs.

### 2.5. Antimicrobial Activity Test

The antimicrobial activity of the synthesized AgNPs was evaluated *in vitro* against specific pathogenic microorganisms using the agar well diffusion method. Mueller–Hinton agar (MHA) was used for bacterial strains, while Sabouraud agar was used to grow *Candida albicans*. The tested microbial strains included *Escherichia coli* (ATCC 93111), *Salmonella typhimurium* (ATCC 14028), *Listeria monocytogenes* (ATCC 25923), *Staphylococcus aureus* (ATCC 25923), and *C. albicans* (ATCC 10231). A sterile cotton swab was used to inoculate standardized bacterial suspensions, which were prepared in sterile 0.85% saline and adjusted to an optical density equivalent to 0.5 McFarland standards (~10^8^ CFU/mL). A sterile gel puncture created wells (6 mm in diameter) on the MHA and Sabouraud agar plates. The AgNPs (30 *μ*g/mL) were carefully introduced into these wells. As a positive control, novobiocin (30 *μ*g/mL) was used for bacterial strains, while amphotericin B (30 *μ*g/mL) served as the positive control for *C. albicans*. The plates were then incubated at 37°C for 24 h for bacterial cultures and at 30°C for *C. albicans*. The antimicrobial activity was assessed by measuring the inhibition zone (in millimeter), and all results were reported as the mean of three independent measurements [18].

### 2.6. Minimum Inhibitory Concentration (MIC) Test

The MIC is the lowest concentration of a chemical or drug that inhibits a bacterium's or bacterial population's visible growth. To determine the MIC of the synthesized AgNPs, serial dilutions of the nanoparticles were performed using the agar diffusion method. Mueller–Hinton broth was used as a diluent for the most sensitive bacterial strain. Approximately 10^6^ CFU/mL bacterial cells were inoculated on MHA plates. The AgNP stock solution (30 *μ*g/mL) was serially diluted up to a concentration of 10^–14^. Following incubation at 37°C for 24 h, the lowest concentration that exhibited inhibition of bacterial growth was recorded as the MIC value. The results presented are the mean of three independent measurements [19].

### 2.7. SEM of Treated Cells

SEM was utilized to examine the structural effects of AgNPs on bacterial cells. For sample preparation, 5 *μ*L of pure AgNPs (0.1 mg/mL) was deposited onto a carbon tape and allowed to air dry at room temperature for 15 min. The dried samples were then analyzed using a HITACHI S-4800 SEM (HITACHI, Japan) to capture high-resolution micrographs of treated bacterial cells, revealing morphological alterations induced by AgNP exposure [20].

## 3. Results

### 3.1. Collection of Leaf Samples

**Figure 1:**
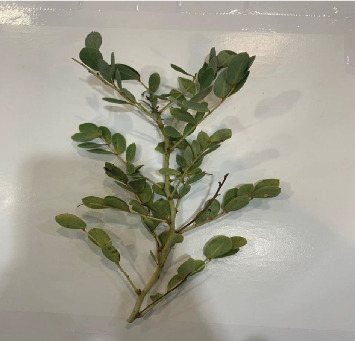
The fresh leaves of *Senna italica*.

Fresh *Senna italica* leaf samples were collected from the Jazan region, KSA, and carefully placed in sterile bags for transportation to the laboratory. Upon arrival, the leaves were thoroughly washed with tap and double-distilled water to remove dust and any surface contaminants. The cleaned leaves were then left to dry in a shaded area to preserve their bioactive compounds before further processing (Figure 1).

### 3.2. Effect of Physical Conditions on the Synthesis of AgNPs

**Figure 2:**
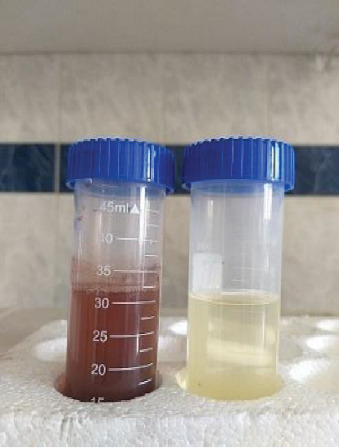
Synthesis of AgNPs by *Senna italica* leaf extract.

The synthesis of AgNPs was influenced by various physical parameters, including extract dilution, extract volume, pH, temperature, and AgNO_3_ concentration. When the plant leaf extract was mixed with AgNO_3_ solution, a gradual color change from pale yellow to dark yellow and finally to colloidal brown was observed, indicating the formation of AgNPs (Figure 2).

#### 3.2.1. Effect of Different Plant Extract Dilutions

**Figure 3:**
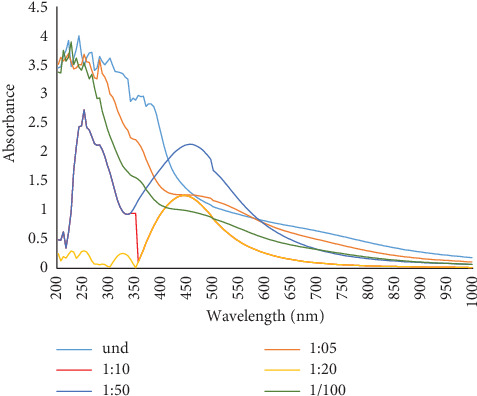
Effect of different leaf extract dilution on the synthesis of AgNPs.

Various dilutions of *Senna italica* leaf extract (1:5, 1:10, 1:20, 1:50, and 1:100) were prepared and mixed with equal volumes of AgNO_3_ solution. The reaction mixture exhibited a color change from pale yellow to brown, confirming the synthesis of AgNPs. UV-Vis spectrophotometric analysis revealed a characteristic absorption peak at 455 nm, confirming the presence of AgNPs. Among the tested dilutions, the 1:50 dilution was found to be optimal for the synthesis of AgNPs, producing the most distinct peak (Figure 3).

#### 3.2.2. Effect of Different AgNO_3_ Concentrations

**Figure 4:**
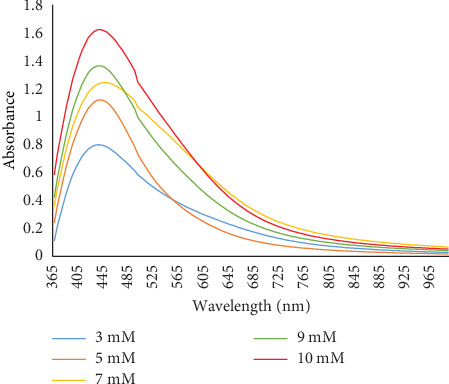
Effect of AgNO_3_ concentrations on the synthesis of AgNPs.

To determine the optimal AgNO_3_ concentration for AgNP synthesis, solutions with varying concentrations (3, 5, 7, 9, and 10 mM) were prepared and mixed with equal volumes of *Senna italica* leaf extract. UV-Vis spectrophotometric analysis showed a peak at 435 nm, which is characteristic of AgNPs. The highest concentration (10 mM AgNO_3_) resulted in the most efficient nanoparticle formation (Figure 4).

#### 3.2.3. Effect of Temperature

**Figure 5:**
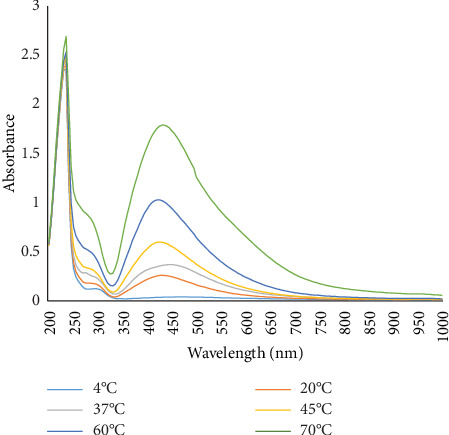
Effect of temperatures on the synthesis of AgNPs.

The influence of temperature on AgNP synthesis was evaluated by incubating the reaction mixture at different temperatures. The highest yield of AgNPs was observed at 70°C, with a UV-Vis spectral absorption peak at 435 nm, confirming the formation of nanoparticles (Figure 5).

#### 3.2.4. Effect of Extract Volume

**Figure 6:**
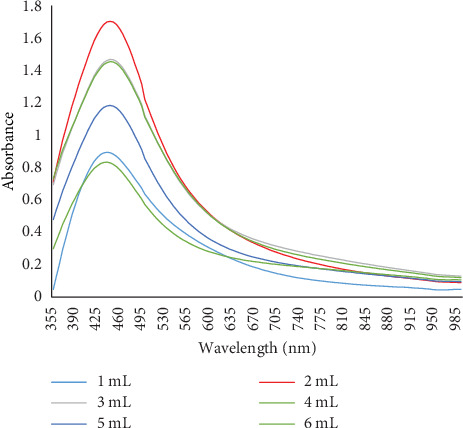
Effect of different leaf extract volumes on the synthesis of AgNPs.

The effect of extract volume on AgNP synthesis was studied by adding varying volumes of *Senna italica* leaf extract (1, 2, 3, 4, and 5 mL) to a 2-mL solution of 10-mM AgNO_3_. The most effective volume for optimal nanoparticle synthesis was found to be 2 mL, as confirmed by a UV-Vis spectral absorption peak around 445 nm (Figure 6).

#### 3.2.5. Effect of pH

**Figure 7:**
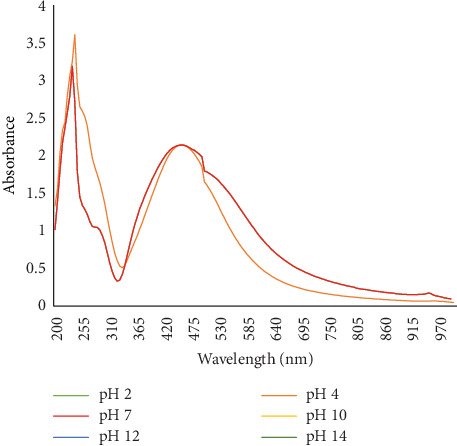
Effect of different pH on the synthesis of AgNPs.

The role of pH in AgNP synthesis was assessed by adjusting the reaction mixture to different pH levels (2, 4, 7, 10, 12, and 14). The formation of AgNPs was monitored, and the results indicated that pH levels of 4 and 7 were the most favorable for nanoparticle synthesis (Figure 7).

### 3.3. Characterization of AgNPs

#### 3.3.1. TEM Analysis

**Figure 8:**
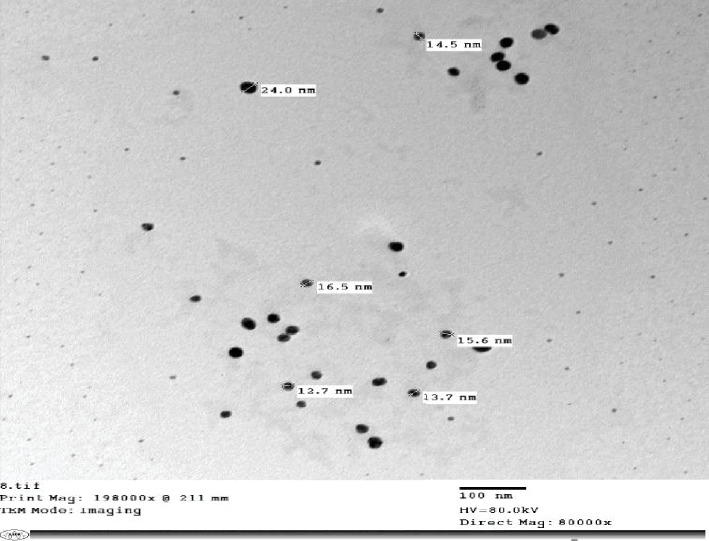
TEM showed the analysis of green synthesized *Senna italica* AgNPs.

TEM analysis was conducted to determine the size, shape, and distribution of the synthesized AgNPs. The micrographs revealed that the nanoparticles were monodispersed and predominantly spherical, with a size range between 12.7 and 24 nm (Figure 8).

#### 3.3.2. XRD Analysis

**Figure 9:**
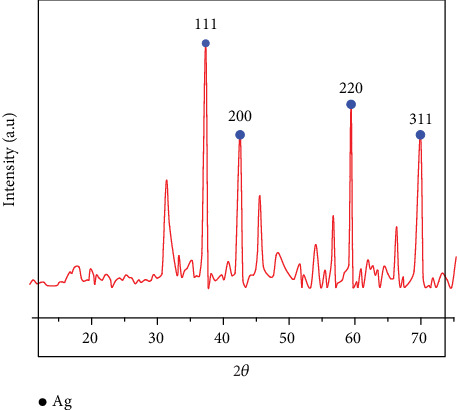
XRD pattern of synthesized silver nanoparticles.

XRD analysis confirmed the crystalline nature of the synthesized AgNPs. Peaks observed at 2*θ* values of 38.0°, 42°, 59°, and 69° corresponded to the (111), (200), (220), and (311) planes of face-centered cubic (FCC) silver, respectively, indicating the presence of crystalline AgNPs (Figure 9).

#### 3.3.3. FTIR Spectroscopy Analysis

**Figure 10:**
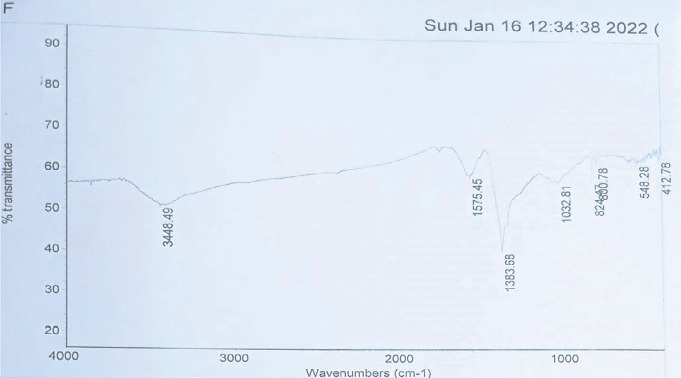
FTIR spectrum of silver nanoparticles synthesized using *Senna italica* leaf extract.

FTIR spectra of the synthesized AgNPs displayed characteristic absorption bands at 412, 548, 800, 824, 1032, 1383, 1575, and 3448 cm^−1^. The peaks at 3448 and 1575 cm^−1^ were associated with hydroxyl (O-H) stretching in alcohols and carbonyl (C=O) groups of aldehydes, ketones, and carboxylic acids, suggesting the involvement of these functional groups in AgNP stabilization (Figure 10).

### 3.4. Antimicrobial Activity of AgNPs

The antimicrobial activity of AgNPs (30 *μ*g/mL) was evaluated against fungal and bacterial strains, including *E. coli* (ATCC 93111), *S. typhimurium* (ATCC 14028), *L. monocytogenes* (ATCC 25923), *S. aureus* (ATCC 25923), and *C. albicans* (ATCC 10231). The inhibition zones recorded were as follows: *E. coli* (13 mm), *S. typhimurium* (11 mm), *L. monocytogenes* (12 mm), *S. aureus* (12 mm), and *C. albicans* (30 mm). These findings confirmed that AgNPs exhibit antimicrobial activity against both prokaryotic and eukaryotic microorganisms.

**Figure 11:**
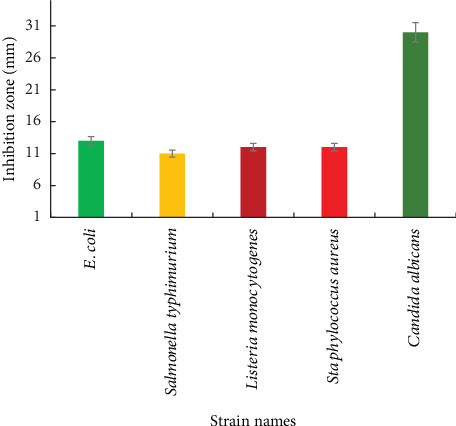
Antimicrobial activity of AgNPs against different microorganisms.

**Figure 12:**
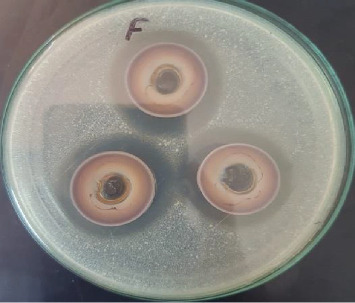
Antimicrobial activity of 30 *μ*g/mL silver nanoparticles.

For comparison, the antibiotic novobiocin showed inhibition zones of 15, 20, 40, and 30 mm against *E. coli*, *S. typhimurium*, *L. monocytogenes*, and *S. aureus*, respectively. Additionally, amphotericin B exhibited an inhibition zone of 20 mm against *C. albicans*. Notably, the highest antimicrobial activity of AgNPs was observed against *E. coli* and *C. albicans* (Table 1 and Figures 11 and 12).

### 3.5. MIC Test

**Figure 13:**
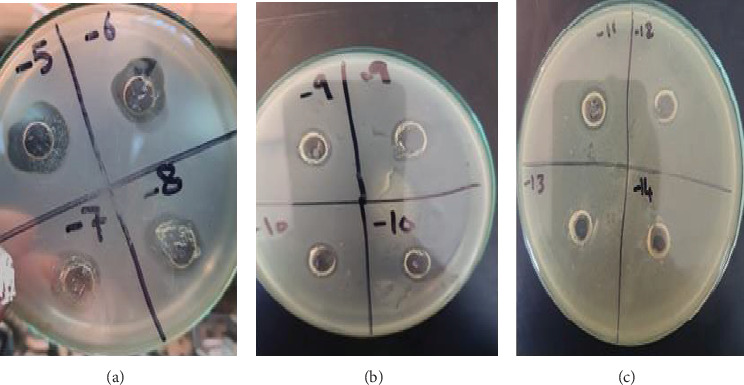
(a–c) MIC test of AgNPs synthesized by *Senna italica* leaf extract against *E. coli* (ATCC93111) at 10^–5^–10^–14^ dilution.

The MIC test was conducted to determine the lowest concentration of AgNPs required to inhibit bacterial growth. The most sensitive strain, *E. coli*, was selected for MIC determination. The results indicated that the MIC for *E. coli* was 0.014 *μ*g/mL at a dilution of 10^–11^, highlighting the potent antibacterial effect of the synthesized AgNPs (Figures 13a, 13b, and 13c).

### 3.6. Effect of AgNPs on Bacterial Cell Morphology

**Figure 14:**
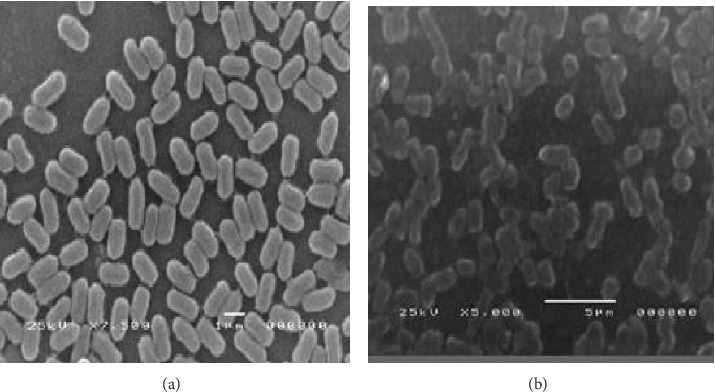
(a) Scanning electron microscopy (SEM) analysis of *Escherichia coli* (ATCC93111) cells before treatment with AgNPs. (b) SEM analysis of *Escherichia coli* (ATCC93111) cells after the treatment with AgNPs.

SEM analysis was performed to investigate the morphological effects of AgNPs on bacterial cells. Significant structural changes were observed in *E. coli* cells treated with AgNPs, where the cells appeared abnormally shrunken and deformed compared to the untreated control cells. The extent of morphological damage was found to be dependent on the concentration of AgNPs applied, further confirming their antimicrobial efficacy (Figure 14a,b).

## 4. Discussion

*Senna* is a significant genus of flowering plants comprising nearly 350 species. The analysis of the leaf extract of *Senna italica* has revealed the presence of flavonoids, alkaloids, and steroids. Additionally, *Senna italica* contains six bioactive compounds: quercetin, rutin, tinnevellin, physcion, and emodin. These bioactive compounds contribute to the medicinal value of *Senna italica*, particularly its antibacterial activity [21, 22]. Various metal nanoparticles, including gold, silver, copper, and platinum, have been extensively studied. However, AgNPs have demonstrated superior antimicrobial efficacy. During the synthesis of AgNPs, the color of the AgNO_3_ solution changed from yellowish to brown upon interaction with the leaf extract [23]. This color change, from yellowish–green to brown, indicates the reduction of ionic silver (Ag^+^) to metallic silver (Ag), which subsequently assembles into colloidal nanoparticles (AgNPs) [24]. Our study identified a UV-Vis absorption peak around 445 nm, characteristic of the plasmon resonance of AgNPs. Previous studies have reported the plasmonic peak of AgNPs synthesized from *Senna alexandrina* to be between 405 and 415 nm [25]. Another study successfully synthesized AgNPs using the bark extract of *Senna alata* [26]. The UV-Vis spectrophotometric analysis of AgNPs showed a peak at 425 nm upon the addition of AgNO_3_ to the plant extract. Similarly, AgNPs synthesized from *Senna hirsuta* displayed maximum absorbance peaks at 436 and 402 nm, respectively [27]. The multifunctionality of *Senna italica*–derived AgNPs stems from their stability, antimicrobial efficiency, and recyclability. Their cyclic stability makes them particularly valuable for environmental and energy applications like wastewater treatment and catalysis. However, further quantitative assessments would provide deeper insights, including stability testing over repeated cycles under real-life conditions. Future studies should incorporate additional experimental data to confirm long-term performance. Interestingly, AgNPs were also synthesized using the leaf extract of *Senna occidentalis*, and their optical absorbance was measured using a UV-Vis spectrophotometer. The results confirmed wavelength values characteristic of metallic AgNPs, approximately 400 nm [28]. A study on the optimization of reaction conditions for AgNP synthesis identified the following optimal parameters: The successful synthesis of AgNPs using plant extracts is influenced by several critical reaction parameters, including extract dilution, AgNO_3_ concentration, extract volume, pH, and temperature. Optimizing these factors ensures the efficient formation of stable, biofunctionalized AgNPs with desirable physicochemical and antimicrobial properties. Plant extracts contain diverse bioactive molecules, including flavonoids, polyphenols, tannins, and proteins, which act as reducing and stabilizing agents during AgNP synthesis. The optimal dilution of 1:50 ensures a sufficient concentration of bioactive molecules for the reduction of Ag^+^ to Ag^0^, prevention of excessive aggregation, which can lead to the formation of larger, nonuniform nanoparticles, and an adequate balance between reducing agents and silver ions, promoting controlled nucleation and uniform growth of nanoparticles. Higher extract concentrations may lead to uncontrolled nucleation, while excessively dilute extracts may not provide enough reducing agents for efficient nanoparticle synthesis. AgNO_3_ serves as the precursor for AgNP formation, as it provides Ag^+^ ions that are reduced by plant-derived phytochemicals. The optimal AgNO_3_ concentration of 10 mM is necessary for a sufficient number of Ag^+^ ions available for reduction, prevention of excessive particle growth or aggregation, and the formation of well-dispersed, monodisperse AgNPs. If the AgNO_3_ concentration is too low, nanoparticle formation may be incomplete. Conversely, excessively high concentrations may result in agglomeration due to excessive nucleation. The volume of plant extract plays a significant role in the synthesis of AgNPs, as it determines the amount of reducing and stabilizing agents available for the reaction. The optimized 2-mL extract volume provides adequate phytochemicals for reducing and stabilizing AgNPs. A balanced reaction environment promotes uniform nanoparticle formation. A lower extract volume might lead to the incomplete reduction of silver ions, while an excessive extract volume may result in unstable nanoparticles or polydisperse particles due to overreduction. The pH is a crucial parameter in AgNP synthesis, affecting the reduction rate, nucleation process, and overall nanoparticle morphology. The optimal pH values of 4 and 7 indicate that, at pH 4 (acidic conditions), the reduction of Ag^+^ ions is slower, leading to controlled nucleation and the formation of smaller, more stable nanoparticles. At pH 7 (neutral conditions), reduction occurs at a moderate rate, allowing for the formation of well-defined nanoparticles with a uniform distribution. Extreme pH conditions (too acidic or too basic) can lead to the instability of nanoparticles, excessive aggregation, or variation in morphology. Moreover, temperature is a key factor influencing the reaction kinetics, nanoparticle formation rate, and stability. The optimized temperature of 70°C promotes faster reduction of Ag^+^ to Ag^0^, leading to rapid nucleation; the formation of smaller, well-dispersed, and crystalline nanoparticles; and enhanced interaction between plant phytochemicals and silver ions, improving capping and stabilization. Lower temperatures may result in slow nucleation, leading to the formation of larger, irregularly shaped nanoparticles. Conversely, extremely high temperatures may cause excessive particle growth and aggregation. TEM analysis revealed AgNPs with a size range of 12.7–24 nm and a predominantly spherical shape. High-resolution TEM images confirmed the formation of spherical AgNPs with an average diameter of 10–30 nm [26]. The AgNPs synthesized using *Senna alexandrina* were reported to have a particle size of approximately 80 nm [29]. SEM analysis of AgNPs synthesized from the flower extract of *Senna siamea* also confirmed a spherical morphology. FTIR spectroscopy analysis confirmed functional groups associated with biomolecules involved in AgNP stabilization. The analysis showed absorption bands at 3448 and 1575 cm^−1^, corresponding to hydroxyl (-OH) stretching in alcohols and carbonyl (C=O) groups of aldehydes, ketones, and carboxylic acids, respectively. AgNPs synthesized from methanolic and ethyl acetate extracts of *Senna occidentalis* exhibited eight prominent peaks in the functional group region. The peaks at 3749, 3272, 2922, 2851, 2098, 1703, 1509, and 1595 cm^−1^ correspond to OH (alcohol), -OH (carboxylic acid), N-H (amine), C-H (alkane), SCN (isothiocyanate), C=O (conjugated aldehyde), and N-O (nitro compounds), respectively. Additional peaks in the fingerprint region suggested the presence of phenols, aromatic amines, and tertiary alcohols [30]. The *Senna alexandrina* leaf extract analysis confirmed the presence of carbohydrates, tannins, saponins, steroids, proteins, flavonoids, glycosides, and alkaloids. FTIR analysis of the AgNPs revealed seven peaks, with one prominent peak around 3309 cm^−1^, likely corresponding to O-H vibrational stretching, hydrogen-bonded hydroxyl groups of phenols, and alcohols. These findings indicate strong interactions between phytochemicals and nanoparticles, suggesting a capping and stabilization effect contributing to AgNP stability and bioactivity [31]. XRD analysis of the synthesized AgNPs confirmed their crystalline nature, revealing FCC silver. The 2*θ* peak values observed were 38.0°, 42°, 59°, and 69°, corresponding to the 111, 200, 220, and 311 planes, respectively. The XRD analysis of AgNPs synthesized from *Senna alata* showed 2*θ* values with corresponding hkl values of 32.18 (002), 37.22 (101), 42.15 (102), 46.17 (110), 34.16 (100), and 52.34 (103). Similarly, for *Senna hirsuta*, the observed values were 28.41 (211), 30.13 (220), 38.55 (311), 40.10 (222), 43.95 (400), 64.22 (440), and 79.60 (533). The observed phase transition from cubic to hexagonal structures upon silver incorporation may be attributed to variations in crystallite size, lattice strain, and interactions with biomolecules from the plant extract during synthesis [32]. The capping and stabilizing agents in the extract likely influenced crystal growth, leading to structural modifications. This phenomenon has been previously reported in studies on biosynthesized AgNPs, where phytochemicals mediate nanoparticle nucleation and growth. The synthesized AgNPs demonstrated antimicrobial activity against both Gram-positive and Gram-negative bacteria and *C. albicans*. Notably, the AgNPs exhibited antibacterial activity against *S. typhimurium*, *S. aureus*, *E. coli*, *L. monocytogenes*, and *C. albicans* [33]. The MIC of the synthesized AgNPs against *E. coli* was recorded as 0.014 *μ*g/mL, suggesting a remarkably high antibacterial efficiency. *E. coli*, a Gram-negative bacterium, possesses an outer membrane composed of lipopolysaccharides (LPS), which often provide resistance to conventional antibiotics. However, AgNPs can penetrate this membrane and disrupt bacterial functions by interacting with sulfur- and phosphorus-containing biomolecules, leading to cell lysis. The exceptionally low MIC value suggests strong bactericidal properties, reinforcing the effectiveness of AgNPs as alternative antimicrobial agents [34]. AgNPs synthesized from *Senna siamea* exhibited MIC values of 15.63 *μ*g/mL against *S. aureus* and 7.81 *μ*g/mL against *E. coli*. These findings indicate that *E. coli* was more susceptible to AgNPs than *S. aureus*, which aligns with previous reports that Gram-negative bacteria are generally more vulnerable to AgNP-induced membrane disruption. This susceptibility is attributed to the relatively thin peptidoglycan layer in Gram-negative bacteria, allowing AgNPs to penetrate the cell membrane and disrupt cellular processes easily. Conversely, *S. aureus*, a Gram-positive bacterium, possesses a thicker peptidoglycan layer, which may offer some resistance to AgNP penetration. However, the observed MIC of 15.63 *μ*g/mL confirms that AgNPs still exert significant antibacterial activity against *S. aureus*, likely by binding to thiol (-SH) groups in bacterial proteins and enzymes, thereby interfering with essential cellular functions [35].

The effectiveness of AgNPs against both Gram-positive (*S. aureus*) and Gram-negative (*E. coli*, *S. typhimurium*, *L. monocytogenes*) bacteria, as well as the fungal pathogen *C. albicans*, highlights their broad-spectrum antimicrobial nature. The mechanism of action against *C. albicans* is likely due to AgNPs disrupting fungal cell walls and interfering with intracellular functions, ultimately leading to fungal cell death. This suggests the potential of AgNPs as antifungal agents, offering an alternative to conventional antifungal treatments, especially in the context of increasing drug resistance [36].

Comparative antibacterial studies showed that *Senna hirsuta*–derived AgNPs exhibited superior antibacterial activity against *Klebsiella pneumoniae*, *Bacillus subtilis*, *E. coli*, *S. aureus*, and *Enterococcus faecalis* compared to those derived from *Senna alata* [37]. The antibacterial effect of AgNPs varies depending on the structure of the bacterial cell wall. Gram-positive bacteria exhibit some resistance due to their thick peptidoglycan layer, whereas Gram-negative bacteria, with thinner cell walls, are more susceptible. AgNPs adhere to bacterial cell membranes, increasing permeability, membrane separation, and lysis. Moreover, AgNPs interact with sulfur-containing proteins, disrupting cellular functions and causing leakage of cytoplasmic contents.

Additionally, AgNPs may inhibit enzymes, denature proteins, and cause DNA damage, leading to bacterial cell death [38].

## 5. Conclusion

This study successfully synthesized green AgNPs using *Senna italica* leaf extract as a reducing and capping agent. The synthesized AgNPs exhibited notable antimicrobial activity, stability, and recyclability. Given the rising global threat of antibiotic resistance, AgNPs present a promising alternative to conventional antibiotics. Future research should focus on elucidating the detailed mechanisms of AgNP interactions with bacterial cells and optimizing their application in medical and environmental fields.

## Figures and Tables

**Table 1:** Antimicrobial activity of AgNPs against different microorganisms.

**Microorganisms**	**Inhibition zone (mm)**	
	**AgNPs**	**Antibiotics**
	**30 *μ*g/mL**	
Bacteria		Novobiocin
*Escherichia coli* (ATCC93111)	13 ± 0.5	15 ± 1.5
*Salmonella typhimurium* (ATCC14028)	11 ± 1.2	20 ± 0.5
*Listeria monocytogenes* (ATCC25923)	12 ± 1	40 ± 1
*Staphylococcus aureus* (ATCC25923)	12 ± 0.8	30 ± 0.7
Fungi		Amphotericin B
*Candida albicans* (ATCC10231)	30 ± 1	20 ± 0.8

## Data Availability

The data that support the finding of this study are available from the corresponding author upon reasonable request.
